# Editorial: Women in sensory neuroscience

**DOI:** 10.3389/fnhum.2023.1252570

**Published:** 2023-07-24

**Authors:** Monica Gori, Alessia Tonelli, Elena Nava

**Affiliations:** ^1^Unit for Visually Impaired People, Italian Institute of Technology, Genoa, Italy; ^2^School of Psychology, University of Sydney, Sydney, NSW, Australia; ^3^Department of Psychology, University of Milano-Bicocca, Milan, Italy

**Keywords:** neuroscience, perception, women in science, sensory processing, sensory deprivation

Women have made remarkable contributions to this field from the early pioneers to the current generation of researchers. Their expertise and dedication have shed light on the intricate workings of the sensory systems, including vision, hearing, touch, taste, and smell. The representation of women in neuroscience has evolved, reflecting significant progress in promoting gender diversity within the field. One notable example is the groundbreaking work of Rita Levi-Montalcini, who, along with Stanley Cohen, discovered nerve growth factor (NGF), and elucidated its role in cell development and survival. Their discoveries laid the foundation for our understanding of neurotrophic factors and their implications in neural development, plasticity, and diseases. Another notable figure is May-Britt Moser, who, along with her husband Edvard Moser and their collaborator John O'Keefe, unraveled the neural mechanisms underlying spatial navigation and discovered the existence of grid cells in the brain. Their work on the brain's inner GPS earned them the Nobel Prize in Physiology or Medicine in 2014. These examples, among many others, highlight the groundbreaking contributions of women in neuroscience, pushing the boundaries of knowledge and transforming our understanding of the brain. Historically, the number of women pursuing careers in neuroscience was relatively limited, facing barriers and biases that hindered their participation. However, in recent decades, women have been actively engaged in various subfields of neuroscience, including sensory neuroscience, cognitive neuroscience, and computational neuroscience. This growth in representation has been fueled by advocacy efforts, mentorship programs, and initiatives promoting inclusivity and equal opportunities. While there is still work to be done to achieve full gender parity, the increasing number of women in neuroscience today demonstrates a positive shift toward a more diverse and inclusive scientific community supporting the importance of continuing efforts to promote gender equality and inclusivity, as it not only benefits women but the entire scientific community and society as a whole. In this Research Topic, we collect contributions from women expert on sensory neuroscience who contributed to a better understanding of important processing in the brain. We have summarized the results of these works considering three main topics that are (1) Low and high levels of sensory processing, (2) Multisensory processing, and (3) Special populations.

## 1. Low and high levels of sensory processing

Perceptual information can be investigated on different levels, from low-level information to more complex processes that develop across the lifespan. Regarding low-level information, Castellotti et al. investigated the role of local information in recognizing occluded images, showing that local information contributes to successfully reconstructing a visual image even when information is severely occluded. In contrast, List showed how selective attention to local and global information eliminates the level-specific priming effect in a multi-modal (audio-visual) context, while this effect has been demonstrated within both the visual and auditory modalities. However, not only low-level processes can influence perception, but also more complex processes involving body ownership and the stage at which people are in their life span. For example, La Rocca et al. found that the sense of ownership over different face identities can be influenced by congruent visuo-tactile and visuo-motor synchronous stimulation. Aloufi et al. demonstrated how sex differences can influence auditory perception. Specifically, following a literature review, the authors highlighted that women have more sensitive hearing than men. However, this higher auditory sensitivity varies concerning the menstrual cycle. Moreover, Incao et al. investigated how aging affects visual perception when faced with a spatial or temporal task, finding a general decline in perceptual acuity in both domains, but that the influence of prior knowledge determined by context does not change. However, more significant inter-individual variability is present in old age, perhaps due to different strategies that older individuals need to adopt to face higher uncertainty in the perceptual process.

## 2. Special population

Part of this Research Topic is dedicated to the role of the senses in promoting the typical development of different aspects of cognitive functions and how the lack of one of these can change the way blind and deaf individuals perform across tasks.

In particular, Leo et al. and Maimon et al. present evidence that congenital visual deprivation does not prevent the development of skills typically dominated by vision, such as object recognition (Leo et al.) and spatial information (Maimon et al.). Indeed, while Leo et al. show that congenitally blind individuals explore objects differently with their hands, but do not present lower accuracy with respect to late blind and sighted controls, Maimon et al. show that congenitally blind can learn to use a sensory substitution system (aimed at supporting spatial navigation) as effectively and as quickly as visually impaired individuals.

Similarly, Buyle and Crollen and Gessa et al. revealed that auditory deprivation does not impair the learning of basic and more complex skills, such as mathematics. Indeed, Buyle and Crollen found comparable subtraction and multiplication skills across deaf and hearing individuals. Gessa et al. found that sound localization in age-related hearing loss can be improved by head-movements, suggesting that self-regulation strategies and active behavior can keep spatial hearing functional.

Finally, Soker-Elimaliah et al. present a work on the relationship between pupil light reflex (PLR) and atypical neurodevelopment. PLR is associated with sensory processing and thus provides a good model to investigate the link between sensory and social functioning, especially in cases when the latter is impaired, such as autism.

## 3. Multimodal

We receive information about the world around us from multiple senses that interact and are combined and integrated into a multisensory framework. In the Research Topics, there is a set of important results related to multisensory processing in typical and atypical individuals considering language, reward, learning, attention, memory, and perceptual processing. In particular, Benetti et al. discussed multimodal processing in face-to-face interactions proposing a neurocognitive model of multimodal face-to-face communication psycholinguistics and sensory neuroscience. Fisher et al. showed that sensory noise might underlie attentional alterations to multisensory integration in a modality-specific manner supporting the idea that attentional paradigm might be used to study sensory processing in neurological disorders. Shvadron et al. applied multisensory knowledge to sensory substitution devices showing that by utilizing a visual-to-auditory sensory substitution device (SSD), the EyeMusic, it was possible to detect shapes by converting images to sound. Antono et al. show that visual and auditory reward cues can produce a value-driven modulation of perception. Finally, Murray and Shams review some recent findings that demonstrate a range of human learning and memory phenomena in which the interactions between visual and auditory modalities play an important role and suggest possible neural mechanisms that can underlie some recent findings.

## Author contributions

All authors listed have made a substantial, direct, and intellectual contribution to the work and approved it for publication.

